# Randomized controlled trial investigating the effectiveness of a multimodal mobile application for the treatment of chronic pain

**DOI:** 10.1080/24740527.2024.2352399

**Published:** 2024-08-19

**Authors:** Cynthia J. Thomson, Hanna Pahl, Luisa V. Giles

**Affiliations:** School of Kinesiology, University of the Fraser Valley, Abbotsford, British Columbia, Canada

**Keywords:** biopsychosocial model, mobile app, pain management, pain education, meditation

## Abstract

**Background:**

Until recently, treatments for chronic pain commonly relied on in-person interventions, and despite more hybrid care options today, capacity for delivery remains challenged. Digital programs focusing on the psychosocial aspects of pain may provide low-barrier alternatives.

**Aims:**

Through a randomized controlled trial, we investigated the effectiveness of a multimodal mobile application.

**Methods:**

Participants (*n = *198; 82% women, mean age = 46.7 [13.1] years; mean pain duration 13.6 [11.2] years) with nonmalignant chronic pain were randomized to either a 6-week intervention (*n* = 98) or a wait-listed usual care group (*n* = 100). The intervention involved regular engagement with a user-guided mobile application (Curable Inc.) informed by the biopsychosocial model of pain that included pain education, meditation, cognitive behavioral therapy, and expressive writing. The co-primary outcomes were pain severity and interference at 6 weeks.

**Results:**

We observed significant improvements in the intervention group compared to the control group with estimated changes of −0.67 (95% confidence interval [CI] −1.04 to −0.29, *P* < .001, *d* = 0.43) and −0.60 (95% CI −1.18 to −0.03, *P* = .04, *d* = 0.27) for pain severity and interference, respectively. There were significant improvements across secondary outcomes (Patient-Reported Outcome Measurement Information System pain interference; pain catastrophizing; anxiety, depression; stress). Frequency of app use was correlated with improved pain interference (*P* < .001) and pain catastrophizing *(P* = 0.018), and changes from baseline persisted in the intervention group at 12 weeks (*P* < .05).

**Conclusions:**

A short-term mobile app intervention resulted in significant improvements across physical and mental health outcomes compared to wait-listed usual care.

## Résumé

**Contexte :** Jusqu'à récemment, les traitements pour la douleur chronique se faisaient principalement en personne. Bien que de plus en plus d'options de soins hybrides soient désormais disponibles, la capacité à fournir ces soins demeure un défi. Les programmes numériques qui se concentrent sur les aspects psychosociaux de la douleur peuvent offrir des solutions de rechange présentant peu de barrières.

**Objectifs :** Dans le cadre d'un essai contrôlé randomisé, nous avons étudié l'efficacité d'une application mobile multimodale.

**Méthodes :** Les participants (n = 198 ; 82 % de femmes, âge moyen = 46,7 [13,1] ans; durée moyenne de la douleur 13,6 [11,2] ans) souffrant de douleur chronique non cancéreuse ont été répartis au hasard entre une intervention de six semaines (n = 98) et un groupe de soins habituels sur liste d'attente (n = 100). L'intervention consistait en l’utilisation régulière d’une application mobile guidée par l'utilisateur, basée sur le modèle biopsychosocial de la douleur comprenant l'éducation à la douleur, la méditation, la thérapie cognitivo-comportementale et l'écriture expressive. Les résultats coprimaires étaient l'intensité de la douleur et l'interférence à six semaines.

**Résultats :** Nous avons observé des améliorations significatives dans le groupe d'intervention par rapport au groupe témoin, avec des changements estimés de -0,67 (intervalle de confiance à 95 % [IC] -1,04 à -0,29, P < 0,001, d = 0,43) et -0,60 (IC 95 % -1,18 à -0,03, P = 0,04, d = 0,27) pour l'intensité de la douleur et l'interférence, respectivement. Des améliorations significatives ont été observées pour les résultats secondaires (interférence de la douleur selon le système d’information sur les mesures de résultats rapportés par les patients; catastrophisation de la douleur; anxiété; dépression et stress). La fréquence d'utilisation de l'application était corrélée à une amélioration de l'interférence de la douleur (P < 0,001) et de la catastrophisation de la douleur (P = 0,018). De plus, les changements par rapport à l'état initial ont persisté dans le groupe d'intervention après 12 semaines (P < 0,05).

**Conclusions :** Une intervention à court terme au moyen d'une application mobile a permis d'améliorer de manière significative les résultats en matière de santé physique et mentale comparativement aux soins habituels sur liste d'attente.

## Introduction

Chronic pain affects approximately one in five Canadians^1^ and is one of the global leading causes of years lived with disability.^2^ Pain becomes chronic when it persists beyond the typical time it would take for tissue to heal and is defined as “an unpleasant sensory or emotional experience” that persists or recurs for longer than 3 months. Chronic pain includes primary chronic pain, which is a condition itself (not better accounted for by an underlying disease), and secondary chronic pain, which is a symptom arising due to an underlying condition.^3^ Subtypes of secondary chronic pain can further be grouped as musculoskeletal, visceral, cancer-related, postsurgical, headache, and neuropathic.^3^ The biopsychosocial (BPS) model of pain suggests that the interconnection of biology with psychological and social factors has an influence on pain.^4,5^ Specifically, pain has been described as a subjective perception of sensory information that can be influenced by genetics, learned experience, psychological state, and sociocultural influences.^5^

Many treatment modalities focus on the sensory or physical aspects of pain, but there has been less emphasis on treating the emotional experience of pain, and pain-specific training for health professionals is lacking.^4^ A systematic review of BPS uptake in clinical practice revealed evidence-to-practice gaps despite the BPS model being in use for well over 20 years.^6,7^ Emerging studies on interdisciplinary interventions that employ the BPS model by addressing the physical, emotional, and cognitive aspects of pain have reported small to medium positive effects.^8,9^ Interventions employing mindfulness meditation training reported small effect sizes for reductions in pain symptoms in mixed chronic pain populations, along with improvements in quality of life, although the quality of evidence analyzed was low.^10^ A meta-analysis on interventions employing neurophysiological pain education, which uses cognitive behavioral techniques (CBT) to reconceptualize beliefs about pain, reported small to moderate effect sizes for pain reduction in patients with chronic lower back pain.^11^ Similarly, moderate to large effect sizes were found in back pain studies employing CBT^12^ or a technique referred to as “pain reprocessing therapy” that aims to shift fear-inducing pain beliefs.^13^

Interventions that focus on pain education, mindfulness training, and CBT do not require physical manipulations and therefore offer remote and digital options for care that are more scalable than physical interventions. A recent meta-analysis reviewed studies on app-based interventions for chronic pain and found small but significant improvements in pain intensity.^14^ Other online or mobile app–based interventions for chronic pain in older persons and adolescents have reported improvements in awareness of pain response,^15^ pain intensity, and emotional functioning.^16^ Multimodal treatments that incorporate the BPS model of pain are recommended, but wait times and geographical hurdles make accessing these multidisciplinary pain centers a challenge.^17^ A multimodal digital tool used in conjunction with usual care may provide a multidisciplinary treatment option not limited by space or geography.^17^

To our knowledge, the mobile app (Curable Inc., www.curablehealth.com) that we assessed, which includes a multimodal approach with several psychological modalities used in pain treatment facilities (e.g., CBT, mindfulness stress reduction, patient education, relaxation/breathing techniques),^17^ has not been independently evaluated to determine its efficacy in the treatment or management of chronic pain. Therefore, the purpose of our randomized controlled trial was to assess the efficacy of a popular mobile app in the treatment of chronic pain. Primary outcomes included pain severity and interference with daily living, and secondary outcomes included pain intensity, beliefs about pain, depression, anxiety, stress, and quality of life. We hypothesized that participants in the intervention group would report a greater reduction in pain severity (Hypothesis 1a) and pain interference (Hypothesis 1b) when compared to usual care participants. Regarding secondary outcomes, we hypothesized that participants in the intervention group would also report improvements in pain catastrophizing, mental health outcomes, and quality of life (Hypothesis 2), and, finally, we expected that changes in pain severity would be correlated with frequency of mobile app usage (Hypothesis 3).

## Materials and methods

### Study design

This parallel-group randomized controlled trial was virtually administered, included international participants, and involved three cohorts enrolled between October 15, 2021, and April 8, 2022. Participants in each cohort were randomized (1:1) to an intervention or wait-list control group. Prior to and following the 6-week intervention (or control), each group completed pre and post questionnaires that measured pain severity, pain intensity, beliefs about pain, emotional states, and indicators of quality of life. Participants provided informed consent through the online survey and the University of the Fraser Valley Human Research Ethics Board in British Columbia, Canada, approved procedures (HREB No. 100820). All procedures are in accordance with the Declaration of Helsinki. The trial was registered with ClinicalTrials.gov prior to participant enrollment (NCT 05090683).

### Participants

#### Participant recruitment

Participants responded to recruitment posts on social media, national and provincial pain newsletters, websites for chronic pain, health center poster boards, and patient recruitment platforms including Reach BC (reachbc.ca) and the National Institutes of Health (clinicaltrials.gov). Recruitment mainly focused on Canadian participants; however, some websites attracted international participants. We aimed to recruit a diverse sample with a range of pain conditions, not limited by a minimum pain severity or participant proximity to our campus given the fully remote and low-barrier nature of the intervention.

#### Participant eligibility

**Figure 1.**
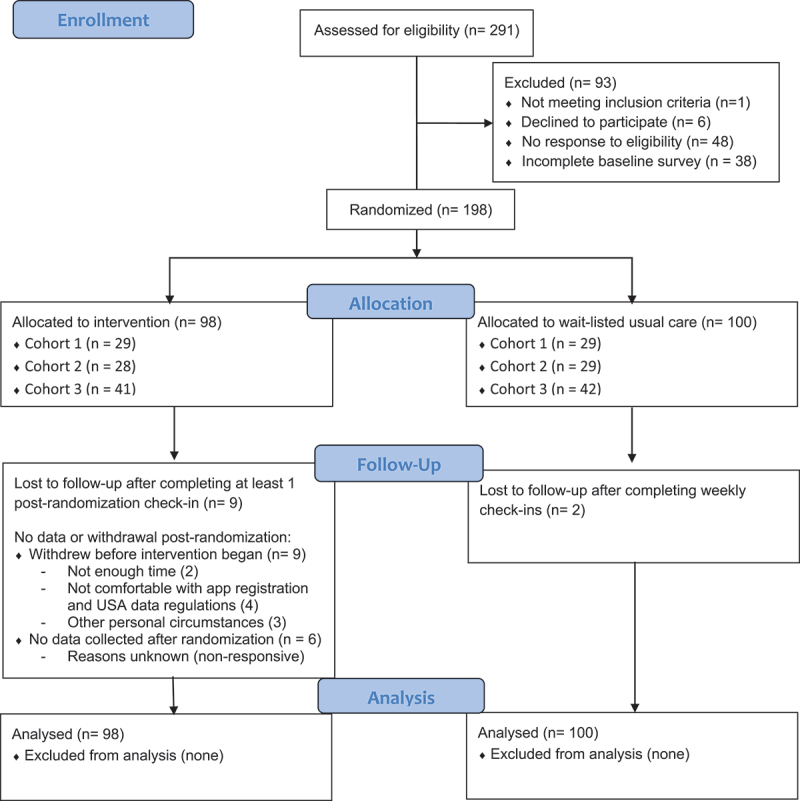
CONSORT [Consolidated Standards of Reporting Trials] flow diagram detailing participant flow through the study.

Participants (*n* = 291) were prescreened for eligibility via e-mail and eligibility was confirmed during the baseline survey using questions to screen for duration and regularity of pain. Of those screened, 93 were not included in the study for the following reasons: not meeting inclusion criteria (*n* = 1), declining to participate (*n* = 6), not responding to eligibility questions (*n* = 48), or not completing a baseline survey (*n* = 38). The remaining 198 participants were randomized to the intervention (*n* = 98) or wait-listed usual care (*n* = 100; Figure 1). Participants were considered eligible if they were 19 – 75 years of age and reported nonmalignant chronic pain for at least 6 months, with pain reported (at any severity) at least every other day.^19^ Participants were excluded if they had current cancer-related pain, had a diagnosed inflammatory condition (such as rheumatoid arthritis, lupus), reported problematic substance use (within 6 months of start date), or reported a psychological condition (e.g., psychotic illness or manic episode) or cognitive impairment that may interfere with program adherence. Participants with previous experience with the mobile app under study were also excluded. Depressive and anxiety disorders were not reasons for exclusion.

#### Participant enrollment

Cohort 1 (*n* = 58) was randomized in October 2021, cohort 2 (*n* = 57) was randomized in January 2022, and cohort 3 (*n* = 83) was randomized in April 2022. Trial recruitment ended following cohort 3 upon reaching the target sample size. Participants gained access to the intervention 1 day following randomization. All data collection occurred remotely via surveymonkey.com.

#### Intervention group

Participants in the intervention group received an access link to the mobile application via e-mail. Within 3 days of group assignment, a research assistant confirmed that participants accessed the app via e-mail. Participants were provided a link to a brief (5-min) orientation video created by C.J.T. to provide an overview of the functions embedded within the app (*https://www.youtube.com/watch?v=QlGTQJvFbvk*). A few participants (*n* = 4) had difficulty gaining access to the app, so we adjusted their survey schedule to start surveys 1 week later. As independent researchers not affiliated with the mobile app under study, the duration of access was limited to the free trial period of the app (6 weeks).

#### Usual care group

Participants in the usual care group were asked to continue with care as usual and completed weekly check-in surveys to record any changes to usual care (including medications or treatments). They were given access to the app following trial completion (at 6 weeks).

### Experimental intervention

The intervention consisted of self-directed use of a personalized (smart) mobile application (Curable Inc). Participants were asked to engage with the app at minimum four times per week but were recommended to attempt daily use. The app provides four types of activities: “education,” which includes audio lessons featuring evidence-based neuroscience education on pain; “brain training,” featuring CBT guided strategies that include both audio and written lessons; “meditation,” which includes pain-specific audio meditations focusing on a variety of topics; and “writing,” which includes exercises that focus on expressive writing in certain situations (e.g., work) or in certain relationships (e.g., parent–child relationships etc. Note: users do not enter their writing or CBT prompts within the app but complete exercises using a writing tool of their choice.). The app has an artificial intelligence interface that tailors activities to the user preferences, which are based on user assessment of the activity usefulness and experience of pain inputted at the start of the app registration. Additional features within the app include two app-affiliated podcasts that include interviews with experts in pain science and patient experiences and another podcast focusing on pain reprocessing therapy^13^ through patient dialogue with a clinical psychologist. Finally, there is a feature for coping with pain in the moment that uses a visual cue to focus on breathing and then provides users with the option of selecting from one of several different techniques (e.g., meditation, visualization, identifying emotions, a pep talk, and/or how to manage a panic attack). Participants were asked to engage with the app by choosing either one of the four activity types or, if time was limited, they were recommended to choose an on-the-go listening option available through the two affiliated podcasts. We did not require them to engage in specific activities because the app is designed to be tailored to user preferences. The Curable app was evaluated for quality of self-management support functions specific to chronic pain and scored the highest of the 19 apps evaluated in both number of self-management functions addressed within the app and scored among the highest in overall app quality as measured by a standardized rating scale (Mobile App Rating Scale).^20^

### Data collection

All participants completed a survey at baseline (pre-randomization) and at the end of the 6-week intervention. An additional follow-up survey was sent only to the intervention group at 12 weeks (the control group did not complete the 12-week survey because they had gained access to the app). Additionally, during the 6-week intervention period, all participants completed weekly check-in surveys to record information from the previous week. The weekly check-in surveys inquired about the frequency of app use (intervention group) and any changes in usual care for both groups (six check-ins). Participants completed surveys remotely and independently using the Survey Monkey website. Survey Monkey sent links directly to participants, which enabled the tracking of missing surveys and allowed for follow-up by our team when necessary. Follow-up reminders were sent within 2 days of sending weekly check-ins and within 5 days for the postintervention surveys. Three attempts at contact by the study team were made before considering the participant a dropout. Participants were removed from the contact list for the weekly check-ins following two consecutive weeks (with three e-mail reminders) with no response and were considered “lost to follow-up.” The final cohort completed data collection by mid-July 2022.

The baseline and 6- and 12-week follow-up questionnaires are outlined in the following section. Two instruments (the Brief Pain Inventory [BPI] and the Patient-Reported Outcome Measurement Information System [PROMIS]), which vary in their recall time (24 h to 7 days) and precision (11-point vs. 5-point), were used to measure ratings of pain severity/intensity and interference with daily living. We included these two commonly used tools for pain, despite redundancy, to align with previous studies. Study surveys also included potential correlates of pain including pain catastrophizing, depression, anxiety, stress, and quality of life as well as demographic characteristics and information on daily and occasional medication use (name of medication, frequency, and dosage).

#### Measures

##### Brief Pain Inventory Short Form

The BPI is a nine-item tool that is used in research and clinical settings (we obtained permission for use through MD Anderson, www.mdanderson.org). It measures pain severity over the last 24 h and at present and measures interference with physical, social, and emotional components due to pain. Each item is scored on 11-point scales ranging from 0 = *no pain* to 10 = *pain as bad as you can imagine* for pain severity items, and 0 = *does not interfere* to 10 = *completely interferes* for interference with activities of daily living.^21^ The single item for pain severity on average, which was a primary outcome in this study, is supported by IMMPACT as a valid clinical outcome.^22^ The mean score of the seven-item pain interference scale (item 9 on the BPI) demonstrated high reliability in our sample (Cronbach’s alpha = 0.90).

##### Patient-reported outcomes measurement information system short form

We used the PROMIS measures for pain intensity (three items, short form 3a), using item 2: “average pain” over the past 7 days and pain interference (sum score of eight items, over 7 days, short form 8a). Both scales use a 5-point Likert scale anchored with 1 = *had no pain* to 5 = *very severe* for intensity, and 1 = *not at all* to 5 = *very much* regarding pain interference.^23^ Scales demonstrated high reliability (Cronbach’s alpha for PROMIS interference = 0.94).

##### Pain catastrophizing scale

The 13-item Pain Catastrophizing Scale (PCS) measures a multidimensional construct that consists of rumination, magnification, and helplessness and has an internal consistency of 0.87 (we obtained permission for use through Mapi Research Trust, http://eprovide.mapi-trust.org).^24^ The scale uses a 5-point Likert scale anchored by 0 = *not at all* to 4 = *all the time* and the total score is a sum of the items (score range 0–52, with higher scores indicating greater catastrophizing). PCS displayed high internal reliability in our sample (Cronbach’s alpha = 0.94).

##### Depression, anxiety, and stress scales

The Depression, Anxiety, and Stress Scale 21 (DASS-21) is a 21-item tool that measures three negative emotional states: depression, anxiety, and stress.^25^ The scale uses a 4-point Likert scale from 0 = *did not apply to me at all* to 3 = *applied to me very much, or most of the time*. The questionnaire enquires about emotional state over the past week, and scores from each subscale (seven items each) can be summed and doubled to obtain a score classification (normal, mild, moderate, severe; subscale score ranges 0–42, with higher scores indicating higher negative emotional state). Overall scale reliability was high (alpha = 0.91), and subscales were acceptable (alphas for each: anxiety = 0.78, depression = 0.89, stress = 0.82).

##### Short form quality of life

The Short Form-12 items (SF-12) measures quality of life using standardized summary scores (0–100) for mental (mental component score, MC-12) and physical (physical component score, PC-12) health and demonstrates construct validity in people reporting pain.^26,27^ Higher component scores indicate greater quality of life.

### Outcomes

Our preplanned co-primary outcomes were the BPI pain severity on average (11-point scale, over 24 h) and BPI pain interference (average score of seven items, 11-point scale) at 6 weeks. Preplanned secondary outcomes included PROMIS pain intensity (over 7 days); PROMIS interference total score; PCS total score; DASS-21 scores for depression, anxiety, and stress; quality of life as measured by the SF-12 mental and physical component scores; and descriptive data on medication use. Preplanned follow-up analyses in the intervention group included correlations between the abovementioned outcomes and frequency of app usage, along with a 12-week follow-up analysis to determine whether changes in the primary and/or secondary outcomes from baseline existed at 12 weeks (the follow-up analyses were detailed in the original National Institutes of Health protocol and research ethics board approved protocol). Finally, we explored the relationship between pain catastrophizing and change in pain severity (not preplanned) through a mediation analysis.

### Sample size

A meta-analysis of mobile app–based interventions for the treatment of chronic pain reported a small effect at Cohen’s *d* = 0.4,^14^ but the content of the apps was highly variable and not all included pain education and/or CBT exercises. Another meta-analysis on psychological interventions that included CBT compared to wait-listed controls reported Cohen’s *d* = 0.62.^12^ Using G*Power software^28^ with an estimated effect size of *f* = 0.3 (or *d* = 0.6), a total sample size of *n* = 90 would be required to achieve 80% power at alpha = 0.05 (powered originally for analysis of covariance [ANCOVA^29^; see data analysis below] but sufficiently powered for linear modeling). We aimed to enroll a minimum of *n* = 120 participants (60 per group). Recruitment following cohort 2 failed to meet our minimum sample; therefore, we recruited a third cohort (*n* = 83), which exceeded our total at our closing date. We opted to include eligible consenting participants from this final cohort to facilitate stratification for randomization.

### Randomization and blinding

After baseline survey completion, 198 participants were stratified and then randomized 1:1 intervention to usual care within each of the three cohorts. Participants were stratified by gender (self-identifying as man, woman, or non-binary) and pain severity score (defined by the BPI pain severity on average in the last 24 h) by the primary investigator (C.J.T.). C.J.T. grouped deidentified data into strata (blinded to all but gender and pain severity scores). There were few men per cohort, so strata for men were created using a median split for pain severity (1–5; 6–10). Owing to the larger number of women in our study, we stratified by BPI severity level (i.e., 4, 5, 6, 7) whenever possible, aiming for minimum 8 participants per stratum to allow for variable block sizes. When the minimum participants per severity level was not achieved (e.g., with BPI severity of 8 or 9), levels were combined to create a stratum that included multiple levels. C.J.T. inputted parameters for a website-generated block randomization to create varying block sizes (2, 4, 8) with two treatment labels that would total to the number of participants in each stratum for randomization (using www.randomization.com). A research assistant (H.P.) received the stratified groups and randomly ordered them before receiving the randomization sequence from C.J.T. H.P. then assigned the interventions per block and per strata on the randomly ordered sample. H.P. notified participants by e-mail; C.J.T. remained blind to the group assignments.

Participants were not blinded and were aware of group assignment. H.P. was not blinded to group assignment and carried out trial administration. All participant contact employed standardized reminders that were identical between groups and were automated and deployed by surveymonkey.com. Follow-up reminders were also preprogrammed through surveymonkey.com. E-mail reminders (if in-program survey reminders failed) were administered by H.P. but were form e-mail reminders composed in advance that were consistent between groups. The researcher (C.J.T.) carrying out the analysis was blinded for the following analyses: the co-primary outcomes of BPI pain severity and interference and for the secondary outcomes of pain catastrophizing, PROMIS pain intensity and interference scores, DASS-21 scores, and SF-12 scores using ANCOVA, which defaults to complete case analysis (see file in Supplemental Digital Content, results from ANCOVA analysis). Given that a more in-depth look into the missing data was warranted following these initial analyses, blinding was removed to assess the nature of the missing values.

### Statistical methods

All analyses were done using IBM SPSS Statistics 25. Our statistical methods changed to account for missing data. A priori we had planned for ANCOVA to compare the effect of the intervention while taking baseline outcome data into account for each group (i.e., baseline data as the covariate, 6-week data as the dependent variable, and group [intervention vs. control] as the independent, between-subject factor).^29^ Following unequal loss to follow-up (deemed missing at random) in the intervention group compared to the control group, a linear mixed model for repeated measures (MMRM) using maximum likelihood (ML) estimation was employed for each outcome to analyze the effectiveness of the intervention at 6 weeks. This method estimates model parameters using all participant data (intention to treat, ITT) without imputation.^30^ Model fixed effects were group, time, and Group × Time, with time as a repeated measure. The best-fitting covariance structure was determined by comparing model fits using the −log likelihood ratio. We report model estimates for main effects and interactions (followed by 95% confidence intervals [CIs]), along with follow-up pairwise comparisons of estimated marginal means (EMM ± SE) when relevant. Statistical significance was set at *P* < .05 for the co-primary outcomes, which are dimensions of the BPI tool (severity and interference), and secondary outcomes (see Outcomes, described above) were significant at *P* < .05. We report adjusted *P* values (*P*_adj_, Bonferroni correction) when reporting EMM differences for pairwise comparisons. We estimated effect sizes based on beta coefficient estimates of fixed effects from MMRM divided by pooled standard deviations for baseline outcomes.^31,32^ We had preplanned to compare descriptive data on medication use at baseline and at 6 weeks using dosage and frequency data, though post hoc we simplified our analysis to include only dichotomous grouping based only on use (“yes” = reported use or “no” = not reported) for the major subclasses of medication reported. We compared frequencies between time (baseline vs. 6 weeks) and groups (intervention vs. usual care) using chi-square (2 × 2) contingency tables. Preplanned follow-up analyses included Pearson correlations (*r*) to measure relationships between primary and secondary outcomes and average app usage. Preplanned follow-up in the intervention group at 12 weeks employed repeated measures *t*-test to determine whether changes in primary and/or secondary outcomes from baseline exist at 12 weeks. Though not preplanned, we carried out exploratory correlation and mediation analyses to determine whether changes in pain catastrophizing contribute to any observed changes in pain severity and interference (informed by findings from Ashar et al.^13^)

## Results

### Participant demographics

Participant demographics are shown in Table 1. The average pain duration for the full sample was 13.6 years (SD = 11.2), ranging from 10 months to 52 years of chronic pain, with most participants identifying as women (82%). Our remotely administered study included participants from nine countries, with the majority residing in Canada (88.8%) and the United States (6%). Our inclusion criteria permitted a range of chronic pain conditions including musculoskeletal and visceral pain, migraine, and fibromyalgia, and we did not distinguish between primary or secondary pain. In the full sample, 69 participants reported receiving a formal diagnosis for a chronic pain condition (35%); of these, 32 had co-occurring diagnoses (46%). Frequencies of the four most reported diagnoses are shown in Table 1. Groups were stratified by gender and pain severity (BPI), and these variables did not differ between groups at baseline. Other variables, including age, pain duration, highest level of education, employment status, and annual household income, did not differ significantly between groups (all *P*s > .05).

**Table 1.** Participant demographics at baseline.

Characteristic	Wait-listed usual care (*n* = 100)	Intervention (*n* = 98)
*Demographic features*		
Age (years), mean(SD)	46.9 (13.0)	46.6 (13.3)
**Gender, *n(%)***		
Woman	84 (84.0)	78 (79.6)
Man	14 (14.0)	15 (15.3)
Non-binary	2 (2.0)	3 (3.1)
Prefer not to answer	0	1.0 (1.0)
**Education, *n*(%)**		
High school or less	10 (10.0)	13 (13.3)
Trade or diploma	33 (33.0)	30 (30.6)
Bachelor’s degree or higher	55 (55.0)	53 (54.1)
**Household income (CAD), *n*(%)**		
< $34 999	27 (27.0)	29 (29.6)
$35 000 to $49 999	3 (3.0)	12 (12.2)
$50 000 to $74 999	20 (20.0)	16 (16.3)
$74 999 to $99 999	20 (20.0)	13 (13.3)
> $100 0000	26 (26.0)	25 (25.5)
**Employment status, *n*(%)**		
Unable to work/on Disability	27 (27.0)	27 (27.5)
Employed (full and part time)	47 (48.0)	50 (51.0)
Other (student, homemaker)	13 (10.0)	10 (10.2)
Retired	13 (13.0)	10 (10.2)
***Clinical features*, mean(SD)**		
Pain duration (years)	14.2 (11.4)	13.0 (11.0)
BPI severity on average	5.4 (1.5)	5.5 (1.6)
**Diagnosis^a^, *n*(%)**		
No diagnosis	64 (64.0.)	66 (67.3)
**Fibromyalgia**	21 (21.0)	19 (19.4)
Myalgic Encephalomyelitis	6 (6.0)^b^	4 (4.1)^b^
Osteoarthritis	7 (7.0)	6 (6.1)
Migraine	13 (13.0)	10 (10.2)
Other (not including above)	7 (7.0)	11 (11.2)
***Medication use n*(%)**^c^		
Analgesics, opioid	30 (30.0)	18 (18.4)
Analgesics, non-opioid	60 (60.0)	63 (64.3)
Anti-depressants	27 (27.0)	29 (29.6)
Cannabinoids	11 (11.0)	9 (9.2)
Antispasmodics	10 (10.0)	12 (12.2)
Anticonvulsants	31 (31.0)	20 (20.4)
CNS depressants	9 (9.0)	6 (6.1)
Other (related to pain)	6 (6.0)	12 (12.2)

*Notes*. Values are *n*(%) for categorical data, mean(SD) for continuous data. BPI = brief pain inventory average severity. CAD = Canadian dollar. Baseline comparisons between groups employed independent *t-*test for continuous variables and *chi* square for frequencies. There were no significant differences between groups for any of the variables.

^a^Counts for diagnoses are not mutually exclusive as many (46% of those with formal diagnoses) reported multiple diagnoses.

^b^All participants reporting myalgic encephalomyelitis also reported fibromyalgia.

^c^Counts for medications are not mutually exclusive as many reported multiple medications.

^d^Counts that do not add up to total group size are missing (participant skipped item).

### Primary outcomes

We carried out an ITT analysis that included all randomized participants (98 intervention, 100 control). We observed differential dropout between intervention (*n* = 25, 25.5%) and control (*n* = 2, 2%) groups, and we determined that the missing data satisfied the criteria for missing at random. Most participants cited lack of time, change of mind, or discomfort with an American-based app or were lost to follow-up, and reasons for dropping out did not appear to be related to the effects of the intervention or to our primary outcomes. There were no differences between baseline BPI average pain severity or interference (*P* > .05) or other demographic variables potentially associated with pain (age, gender, education, income; *P > *0.05) between completers and noncompleters in either group. There were no missing data for the completers for our co-primary outcomes (0%; or for the PROMIS pain measures). We report the data from linear mixed model analysis below. Data from our blinded analyses that employed ANCOVA for complete case data (73 intervention, 98 control) were consistent with these findings and are reported in our supplementary data (see file in Supplemental Digital Content).

#### BPI pain severity

**Figure 2.**
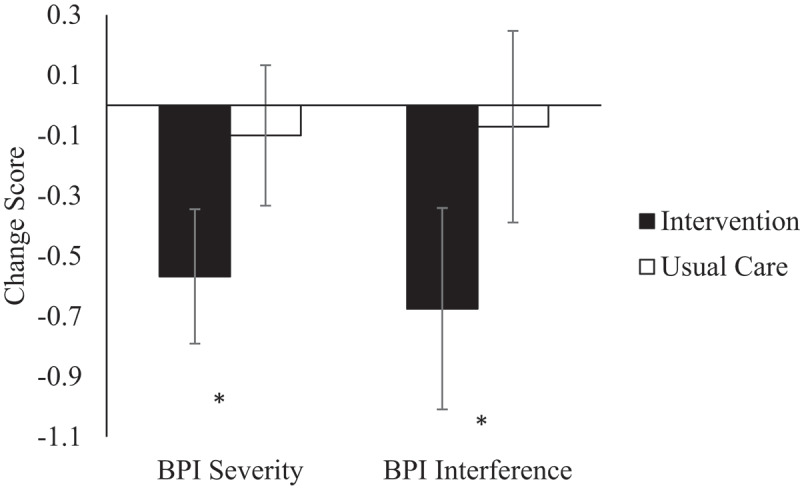
Mean difference based on estimated marginal means from linear mixed modeling of ITT sample for BPI primary outcomes from baseline to 6 weeks (*n* = 98 for intervention, *n* = 100 for usual care). BPI severity reflects pain on average over the past 24 h. BPI interference is an average score. *P < .05. Error bars represent standard error of estimated mean difference. *P* values are adjusted for multiple comparisons using Bonferroni correction.

We checked assumptions for linear mixed modeling. Residuals were normally distributed. There was one outlier (studentized residual >3, at 3.18), but given that the outlier appears to be an unusual value (pain severity increased by 3 points) but does not appear to be due to error, we retained the data point. We carried out linear MMRM using ML estimation. There was no difference between the models employing unstructured (UN) versus compound symmetry (CS) covariance structure (based on −2log likelihood), so we selected the simpler model (CS, based on fewer parameters). There was a significant main effect of time on pain severity as measured by the BPI average score in the past 24 h, *F*(1, 175.7) = 32.70, *P* < .001, but the main effect of group was not significant (*P* = 0.26). This was qualified by a significant interaction of time by group, *F*(1, 175.7) = 12.45, *P* < .001, parameter estimate of −0.67 (95% CI −1.04 to −0.29, estimated Cohen’s *d* = 0.43). After adjusting for multiple contrasts, BPI pain severity was lower following the intervention compared to the usual care group by EMM difference of −0.57 (95% CI −1.03 to −0.11, *P*_adj_ = 0.015; Table 2, Figure 2). Considering data from complete cases at the 6-week follow-up, 20.4% of individuals in the intervention group reported a minimal meaningful pain reduction of 2 points on the numerical rating scale^22^ (20/98) compared to 10.0% of participants from the usual care group (10/100).

**Table 2.** Estimate marginal means for all outcomes.

	Usual care group, *n* = 100		Intervention group, *n* = 98		Between subjects time by group		Intervention group, *n=* 65	Within subjects
Outcome	Baseline	6-weeks	Baseline	6-weeks	*p^a^*	*d^b^*	12 weeks^c^	Baseline to 12 weeks *p^d^*
BPI severity	5.40 ± 0.16	5.19 ± 0.16	5.50 ± 0.16	4.62 ± 0.17	.**001**	0.43	4.60 ± 0.21	**<.001**
BPI interference	6.11 ± 0.22	5.54 ± 0.23	6.04 ± 0.23	4.86 ± 0.25	.**038**	0.27	4.85 ± 0.31	**<.001**
PROMIS intensity	3.22 ± 0.06	3.08 ± 0.06	3.07 ± 0.06	2.85 ± 0.07	.36	0.14	2.74 ± 0.07	**<.001**
PROMIS interference	29.11 ± 0.78	19.70 ± 0.78	28.69 ± 0.78	17.05 ± 0.85	.**013**	0.30	24.34 ± 0.93	**<.001**
Pain catastrophizing	23.48 ± 1.17	20.63 ± 1.18	22.97 ± 1.18	16.22 ± 1.28	.**005**	0.33	16.46 ± 1.59	**<.001**
DASS-depression	15.06 ± 1.00	15.26 ± 1.01	14.82 ± 1.02	12.16 ± 1.09	.**008**	0.59	12.65 ± 1.24	.**042**
DASS-anxiety	9.30 ± 0.75	9.75 ± 0.78	9.91 ± 0.76	7.35 ± 0.84	.**001**	0.79	8.31 ± 0.92	.**013**
DASS-stress	15.62 ± 0.85	15.63 ± 0.86	16.93 ± 0.86	13.99 ± 0.94	.**009**	0.69	15.17 ± 1.56	.**026**
SF-12 PC	21.58 ± 1.05	23.88 ± 1.06	24.42 ± 1.06	28.68 ± 1.14	.11	0.18	27.66 (10.34)	.**005**
SF-12 MC	48.67 ± 0.66	49.70 ± 0.67	47.83 ± 0.67	49.22 ± 0.73	.66	0.06	50.20 (7.62)	.**005**

*Notes*. Values are estimated marginal mean ± standard error based on linear mixed model repeated measures. BPI = brief pain inventory, PROMIS = patient reported outcome measurement information system, DASS = depression, anxiety, stress scale, SF-12 = short form quality of life scale, PC = physical component score, MC = mental component score. Values for the DASS-21 have been doubled to be comparable with the full length DASS-42. Values reported for BPI pain severity and PROMIS pain intensity reflect participant rating for pain severity/intensity “on average”.

^a^*p* value for the time by group interaction from tests of fixed effects. Significant values are shown in bold.

*^b^d* = Cohen’s *d* estimated from parameter estimates for fixed effect interaction divided by pooled standard deviation at baseline.

^c^values are for participants from the intervention group that completed an additional follow-up at 12 weeks. 12-week data is complete and not estimated.

^d^*p* value for the within-group differences (paired *t*-test) in the intervention group only (baseline to 12 weeks).

#### BPI pain interference

BPI interference residuals were normally distributed. The best-fitting model employed ML estimation with a CS covariance structure (model criteria did not differ UN vs. CS, so the simpler model was employed). The main effect of group was not significant (*P* = 0.20), but there was a significant main effect of time, *F*(1, 181) = 37.02, *P* < .001, because both groups showed improved interference scores. This was qualified by a significant interaction of Time × Group, *F*(1, 181) = 4.35, *P* = 0.038, parameter estimate of −0.60 (95% CI −1.18 to −0.03, *d* = 0.27), with a greater improvement (decrease) in BPI interference in the intervention group compared to the usual care group (Table 2, Figure 2).

#### Trial adherence

Trial adherence for trial completers based on guidelines provided to participants in terms of frequency of app use was good. Most participants engaged with the app an average of four times per week (56 of the 73 completers, 77%), and when prescribed weekly minimum engagement was considered, 43 reported 100% compliance (56%; at least four times weekly for full 6 weeks), and 52 reported meeting the minimum engagement on 5 of the 6 weeks of the intervention (71%). With regards to maintaining usual care, two participants each in the intervention and usual care groups reported having surgeries on regions that were reported as a pain region at baseline. We elected not to carry out per protocol analyses as recommended to avoid bias in deciding on exclusions.^33^

### Secondary outcomes

The ITT analysis of secondary outcomes revealed significant positive effects of the intervention (compared to usual care) for pain catastrophizing, PROMIS interference, and the DASS-21 subscales for depression, anxiety, and stress. There were minimal missing data overall for trial completers (e.g., for pain catastrophizing [PCS] a total of 11 data points missing out of 4446 data points, or <0.01%). Given that a single missing item in a sum score leads to a lower total score, we imputed the individual’s mode score when applicable. When an individual was missing three or more points from a single subscale in the DASS (7 items each) the score was not summed. Finally, for the SF-12 we imputed weighted mean scores (total missingness <0.01%).

#### Pain catastrophizing

There was one outlier (studentized residual >3, at 3.08), and data were normally distributed at baseline but slightly skewed at 6 weeks. Results were largely the same with or without the outlier, so we opted to retain the outlier. The best-fitting model employed ML estimation with a CS covariance structure (fewer parameters, no differences in model criteria). There was a significant main effect of time, *F*(1, 177.2) = 50.08, *P* < .001, because both groups showed improved (decreased) PCS scores, and the main effect of group was not significant (*P* = 0.12). There was a significant Time × Group interaction, *F*(1, 177.2) = 8.26, *P* = 0.005, parameter estimate of −3.90 (95% CI −6.58 to −1.22, *d* = 0.33), such that there was a greater decrease in PCS scores in the intervention group compared to the usual care group (Table 2).

#### PROMIS

PROMIS pain intensity on average scores were normally distributed, with no outliers. The best-fitting model employed ML estimation with a CS covariance structure (fewer parameters, no differences in model criteria). The PROMIS intensity scale uses a 5-point scale with a 7-day recall period. Main effects for time and group were significant, whereas the intervention did not result in improvement over the usual care group (interaction of Time × Group was not significant, *P* = 0.4). Both groups improved over time, *F*(1, 180.8) = 15.0, *P* < .001, parameter estimate of −0.14 (95% CI −0.26 to −0.01, *d* = 0.22; EMM shown in Table 2), and the intervention group reported lower scores, *F*(1, 197.0) = 6.15, *P* = 0.014, parameter estimate of −0.23 (95% CI −0.41 to −0.05, *d* = 0.39).

The second PROMIS scale that we employed measures interference with daily living. There were no outliers, and a visual inspection of residuals revealed that the data were normally distributed at baseline but platykurtic (intervention group) and slightly skewed (usual care group) at 6 weeks. Given the robustness of MMRM for violations of normality, we proceeded with the analysis.^33^ The best-fitting model employed ML and an unstructured covariance (based on −2log ratio). There was a main effect of time, *F*(1, 172.5) = 547.13, *P* < .001, and this was qualified by a significant interaction (Time × Group; *F*[1, 172.5] = 5.97, *P* = 0.016), parameter estimate of −2.19 (95% CI −3.94 to −0.42, *d* = 0.28). Both groups improved over time, but the intervention group improved to a greater extent (Table 2). The main effect of group was not significant for PROMIS interference (*P* = 0.2).

#### DASS-21

For each of the DASS-21 subscales, the best-fitting model employed ML and covariance structures did not differ between UN and CS, so the simpler CS model was used for each.

##### DASS anxiety

Data from the DASS-21 subscales were skewed and there were two outliers for DASS anxiety (residuals >3, both at 3.19). Results were consistent with and without the inclusion of the outliers, and given that these did not appear to be an error (but a greater increase in anxiety compared to others who decreased), we retained the cases. MMRM analysis (ML, CS) revealed that there was a main effect of time, *F*(1, 174.6) = 6.18, *P* = 0.014, with decreased anxiety from start to end of trial, with no main effect for group (*P* = 0.39). This was qualified by a significant interaction, *F*(1, 174.6) = 12.38, *P* = 0.001, parameter estimate of −3.00 (95% CI −4.70 to −1.32, *d* = 0.79), with greater reductions in anxiety in the intervention group (EMM in Table 2).

##### DASS depression

Data for the depression subscale were normally distributed and there were no outliers. Mixed modeling (ML, CS) revealed a significant main effect of time, *F*(1, 173.3) = 5.25, *P* = 0.023, and this was qualified by a significant Time × Group interaction, *F*(1, 173.3) = 7.13, *P* = 0.008, parameter estimate of −2.86 (95% CI −4.98 to −0.75, *d* = 0.59), and there was no main effect for group (*P* = 0.2). Over time, both groups improved, though participants reported lower scores in DASS depression following the intervention compared to usual care (EMM in Table 2).

##### DASS stress

Data for the final subscale of the DASS-21, DASS stress, had no outliers (>3) but the data were skewed. Given the robustness of MMRM for violations of normality, we proceeded with the analyses.^34^ Mixed modeling (ML, CS) analysis revealed no main effect of group (*P* = 0.9) but a significant main effect of time, *F*(1, 176.9) = 6.88, *P* = 0.009, that was qualified by a significant interaction of Time × Group, *F*(1, 176.9) = 6.94, *P* = 0.009, parameter estimate of −2.95 (95% CI −5.16 to −0.74, *d* = 0.69). Both groups improved over time (lower scores), though the intervention group improved more than the usual care group (Table 2); following Bonferroni correction the pairwise comparisons for the interaction were not significant (*P*_adj_ = 0.2).

The moderate to large effect sizes for DASS anxiety, depression, and stress are all considered clinically important as per IMMPACT recommendations.^22^

#### SF-12 quality of life

Both the physical and mental component summary scales (PC-12, MC-12) for the SF-12 satisfied assumptions for MMRM. The best-fitting model for the PC-12 (physical) employed ML and an unstructured covariance (based on −2log ratio) and revealed significant main effects for time, *F*(1, 170.4) = 31.38, *P* < .001, with a parameter estimate for time of +2.29 (95% CI 0.79 – 3.80, *d* = 0.22). Main effects of group were also significant, *F*(1, 200.8) = 7.10, *P* = 0.005, parameter estimate of +4.73 (95% CI 1.48 – 7.97, *d* = 0.46). The interaction was not significant (*P* = 0.1). Both groups improved (increased scores) over time, and the intervention group reported higher scores for the physical component scale compared to the usual care group (Table 2).

For the mental component score for the SF-12 the covariance structures did not differ between UN and CS; therefore, the simpler CS model employing ML estimation was used. There were significant main effects for time, *F*(1, 172.8) = 8.74, *P* = 0.004, parameter estimate of +1.03 (95% CI −0.04 – 2.11), which did not reach significance in the estimated model (*P* = 0.06, *d* = 0.16), whereas group and the interaction Time × Group were not significant (*P* > .45). Participants in both groups had improved mental health scores over time. We note that both groups reported physical component scores well below population mean (below the 25th percentile of the general U.S. population), and mental component scores fell around the mean in both groups.^35^

#### Medication use

We had preplanned a comparison of medication use across the 6 weeks using descriptive data on frequency/dosage, but given the wide array of medications and dosages disclosed by our sample, we instead present a simplified frequency summary in our baseline data (Table 1) for the full ITT sample and provide frequency data for baseline and 6 weeks between groups for study completers (Supplemental Table S3). At baseline, the most used medications in the full sample (with reported use within the last 24 h or past 7 days) were nonopioid analgesics (or antihyperalgesics; 63%), followed by antidepressants (27%), anticonvulsants (26%), and opioid analgesics (23%). Eleven percent of the total sample reported no regular use of medications for the treatment of pain. At baseline (ITT sample), the two groups did not differ in their frequency of regular use or in the subclasses of medication usage (Table 1, all *P*s > .05). The data for completers revealed no differences in medication usage between groups at baseline, save reported use of anticonvulsant medications in the usual care vs. intervention group (31.6% vs. 17.8%, χ^2^= 4.18, *P* = 0.041). The only within-group change over time was in the usual care group, with a reported increase in the use of nonopioid analgesics from baseline to 6 weeks (60.2 vs. 73.5%, χ^2^ = 3.89, *P* = 0.049). Finally, between groups at 6 weeks there was a difference in central nervous system depressant use, with the usual care group reporting greater use at 6 weeks compared to the intervention group (9.2% vs. 1.4%, Fisher’s exact test: *P* = 0.029), with no difference between groups at baseline (9.2% vs. 8.2%, *P* = 1.0). If we consider multiple testing and a corrected threshold for significance, none of the changes in medication usage would be considered significant.

### Secondary analyses

#### App frequency

We explored whether frequency of app usage was associated with changes in outcomes (preplanned analysis). This analysis included complete cases (*n* = 73) that had provided frequency of app usage throughout the trial. Although there was no significant correlation between average app usage and change in one of our co-primary outcomes, BPI pain severity (*P* = 0.2), there were significant negative correlations with changes in pain catastrophizing, *r*(73) = −0.28, *P* = 0.018, and BPI interference, *r*(73) = −0.42, *P* < .001. There were no significant correlations with any of the DASS subscales (*P* > .05). Finally, the physical component score for SF-12 was positively correlated with app usage, *r*(73) = 0.27, *P* = 0.02, but not with the mental component score (*P* = 0.08). The significant correlations described above indicate that higher app usage was associated with improvements in these outcomes.

#### Mediation analysis

There were significant correlations between changes in our co-primary outcomes (BPI pain severity, BPI pain interference) and changes in pain catastrophizing in the intervention (BPI severity: *r*[73] = 0.384, *P* < .001; BPI interference: *r*[73] = 0.536, *P* < .001) and usual care (BPI severity: *r*[96] = 0.223, *P* = 0.029; BPI interference *r*[96] = 0.376, *P* < .001) samples. We explored (not preplanned) whether changes in pain catastrophizing (PCS) mediated treatment effects on BPI pain severity and pain interference using linear regression models to estimate effects of PCS as a mediator (using complete case data). We also explored the reverse: whether treatment effects on pain catastrophizing were mediated by changes in BPI scores. The indirect effects between changes in PCS scores and changes in BPI scores (severity and interference) were both significant based on Sobel’s test^36^ (all *P*s < .05). See Figures 3a and 3b for path analysis. The reverse path indirect effects exploring BPI pain severity as a mediator for pain catastrophizing was also significant (*P* < .01; data not shown), whereas the indirect effects for changes in BPI interference as a mediator for PCS were not significant (*P* = 0.052).

**Figure 3a.**
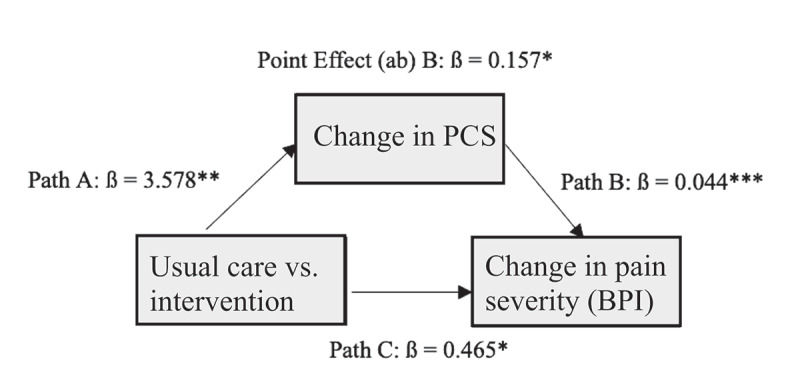
Mediation analysis for the effect of pain catastrophizing (PCS) on changes in BPI average pain severity using complete case data. Point effect is the estimate of the indirect effect between group (usual care ***vs***. intervention) and pain intensity through changes in the PCS at *P* = 0.027 (using Sobel’s test). *P < .05, **P < .01, ***P < .001.

**Figure 3b.**
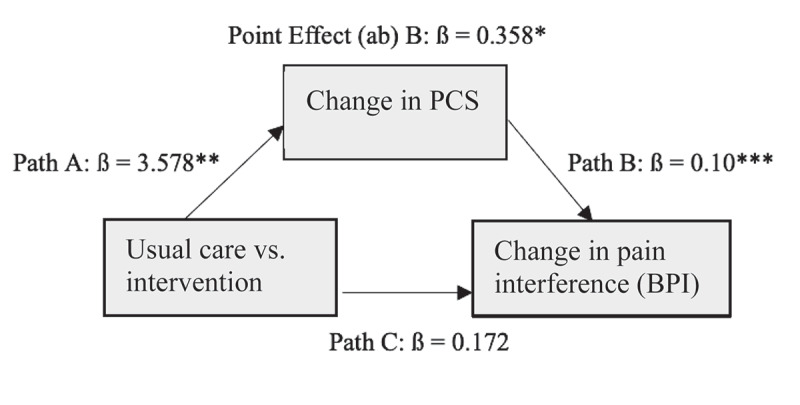
Mediation analysis for the effect of pain catastrophizing (PCS) on changes in BPI pain interference using complete cases. Point effect is the estimate of the indirect effect between group (usual care vs. intervention) and pain interference through changes in the PCS at *P* = 0.013 (Sobel’s test). *P < .05, **P < .01, ***P < .001.

#### 12-Week follow-up

There was a high response rate (*n* = 65 [89%] of 73) for the postintervention follow-up at 12 weeks. Most participants reported having discontinued app usage at the termination of the free trial (*n* = 55; 6 weeks). The 10 participants who continued using the app reported an average frequency of 4.1 times per week and reported a mean change of −1.8 ± 1.87 (*d* = 0.96) for BPI severity at 12 weeks. The wait-listed controls had gained access to the app and were released from the study (we did not track their app usage); therefore, analyses include only the intervention group (no between-group comparisons). Dependent *t*-tests comparing 12-week outcomes to baseline (preplanned analysis) showed significant improvements from baseline values in several outcomes. Primary outcomes were BPI severity, *t*(64) = 4.256, *P* < .001, *d* = 0.53, and BPI interference, *t*(64) = 4.665, *P* < .001, *d* = 0.58. Secondary outcomes were PCS, *t*(64) = 5.879, *P* < .001, *d* = 0.73, DASS anxiety, *t*(63) = 2.544, *P* = 0.013, *d* = 0.318, DASS depression, *t*(64) = 2.074, *P* = 0.042, *d* = 0.28, and DASS stress, *t*(63) = 2.273, *P* = 0.026, *d* = 0.28. The PROMIS measures for pain intensity and interference over 7 days were also significant (intensity: *t*[64] = 3.737, *P* < .001, *d* = 0.46; interference: *t*[64] = 5.128, *P* < .001, *d* = 0.64). Finally, the SF-12 quality of life component scores were significant (PC-12: *t*[64] = −2.893, *P* = 0.005, *d* = −0.36; MC-12: *t*[64] = −2.918, *P* = 0.005, *d* = −0.36).

### Adverse events

No adverse events were reported.

## Discussion

A 6-week trial using the mobile app–based psychological intervention Curable Inc. resulted in significant improvements in several pain- and mental health–related outcomes compared to a wait-listed control group. Results were consistent between ITT analysis and the complete case analysis. Significant improvements from baseline were also observed at the 12-week follow-up in the intervention group. The level of engagement in the app (based on frequency of use) was associated with changes in pain outcomes of pain interference, pain catastrophizing, and the physical component of the SF-12. Pain catastrophizing mediated the relationship (bidirectionally) between the assigned group (intervention vs. usual care) and changes in pain severity and pain interference.

On average BPI pain severity was 0.67 points lower following a 6-week intervention compared to the usual care group, which represents a small effect size (estimated effect size calculated from interaction coefficient from MMRM outcome *d* = 0.43).^31,32^ Our results are in line with others who found that remotely administered multidisciplinary interventions using mobile apps or online platforms resulted in lower pain intensity or severity with similarly small to medium effect sizes in mixed chronic pain populations.^14,16,37,38^ This contrasts with an internet-based intervention that reported improvements in pain severity over time with no differences between groups in samples with musculoskeletal (osteoarthritis) pain.^39^ Though the changes in pain severity and interference with daily living in our study are relatively small (<1 point), improvements over usual care are encouraging because the intervention was relatively brief (6 weeks), and improvements (of moderate size, based on Cohen’s *d*) persisted at 12 weeks despite most participants discontinuing app usage. When compared with a longer (12-week) multidimensional intervention in patients with musculoskeletal chronic pain that included optimized analgesic therapy, CBT and self-management strategies supported by physicians and biweekly contact with research nurses,^40^ the magnitude of pain severity and interference improvements in our study and the former is comparable. A mobile app, such as the one evaluated in our study, represents a more affordable approach to chronic pain management. Furthermore, given that wait times to access pain clinic services can take months to years,^17,41^ an app-based approach that yields similar results to in-person programs represents a more scalable treatment option.

Ashar et al.’s^13^ in-person pain reprocessing therapy (PRT) intervention for back pain reported large effect sizes for changes in pain outcomes, which is rare for psychological interventions. PRT and the Curable app share some techniques such as reappraisal of pain sensations (referred to as “somatic tracking”), exploring difficult emotions that may be associated with pain, and practicing self-compassion and positive self-talk, and both interventions begin with education about the brain’s role in pain. The current study reports much smaller effect sizes compared to those of Ashar et al.,^13^ and though the Curable app includes a podcast that focuses on pain reprocessing along with several brain training exercises (guided by one of the authors from the PRT trial), we cannot determine which participants in our study engaged with these exercises because the app is user-guided and we did not require activity tracking in this study. Further, our study included a heterogeneous chronic pain sample, whereas the former included back pain only.

We observed changes in both pain severity and interference with activities of daily living and subjective feelings associated with pain. The intervention had a positive effect on participants’ beliefs about their pain based on scores from the PCS. The app is informed by the idea that retraining the brain (e.g., using CBT) to perceive pain differently can reduce symptoms and allow the person to gain more control over their pain (Curable Inc.)^18^, and many of the activities embedded within the app relate to repetitive thoughts about pain (i.e., rumination), changing the valence in how we interpret signals of pain (i.e., magnification), and empowering the individual to take control over their pain (i.e., helplessness), each of which are subdomains of pain catastrophizing.^24^ Based on the changes we observed in the PCS scores, along with mediation analyses reported in a previous trial,^13^ we included a post hoc mediation analysis to explore the influence of PCS on pain severity (and the reverse). Thoughts and beliefs about pain as measured by the PCS have been shown to be predictive of pain intensity following induced injury,^42^ and pain catastrophizing is considered a potent predictor of several pain-related outcomes.^43^ In the current study, PCS scores mediated the treatment effects on pain severity and interference, and this mediation effect was bidirectional for pain severity, with pain severity mediating the treatment effects on pain catastrophizing. Similarly, pain beliefs as measured by the Tampa Scale of Kinesiophobia mediated treatment effects on pain severity in a bidirectional fashion in the PRT study mentioned above.^13^

Other notable changes in the current study include moderate (Cohen’s *d* > .5) improvements in emotional states, with intervention group reporting significantly lower scores in depression, anxiety, and stress compared to wait-listed controls, with the strongest effect for anxiety (*d = *0.79). Depression, and especially anxiety, are comorbid factors commonly associated with chronic pain,^44^ and given that anxiety can result in increased avoidance, which can further exacerbate pain,^45,46^ a reduction in anxiety level may have a beneficial effect on pain outcomes. The Curable app includes a variety of meditation audio scripts that include topics related to self-compassion, past traumas, identifying emotions, and guided relaxation. Meditation practice has been shown to improve depression and quality of life, along with pain severity, in a variety of pain samples,^10^ which is consistent with our results. Mindfulness meditation commonly aims to refocus the mind on the present moment, encourage openness and nonjudgment of thoughts, and includes practice of attentional control and diaphragmatic breathing.^10,47^ Interventions that include an emphasis on diaphragmatic breathing have been shown to improve mindfulness, attentional control, and symptoms of anxiety, depression, and stress in healthy adults^48,49^ and in women with fibromyalgia.^50^ Similarly, regular practice of expressive writing has a small, but significant, effect on reducing symptoms of depression, anxiety, and stress.^51^ Curable has expressive writing activities embedded within the app that include identification of stressors, emotional triggers, releasing emotions, and reflections on social relationships. Such exercises may provide insight into social and emotional triggers associated with pain.^52^ Working through emotional events with writing exercises may improve emotional regulation and decrease rumination, which may be particularly important for participants scoring high on PCS or for individuals who have a tendency to suppress emotions.^53^ Overall, the intervention resulted in moderate to large improvements in emotional states, which may also allow for increased optimism, improved attentional control, and the use of active coping strategies (over passive strategies).^54^

An optimistic tone is present throughout the Curable app (and in the name of the app itself), and some of the messaging within the app suggests that recovery from chronic pain is a possibility, and pain-free outcomes have been reported following psychologically focused interventions.^13,55^ There are several anecdotal stories that can be accessed through the app (“recovery stories”), which may provide participants with a sense of hope as they learn about experiences from other patients with similar conditions. Tankha and colleagues^55^ delved into patient experiences from the PRT intervention, and one patient-reported mechanism included peer models of recovery that may have generated hope in the patients.^13^ There is scant literature on the relationship between hope and chronic pain,^56^ but the few studies that exist have shown that hope is negatively associated with pain catastrophizing^57^ and psychological distress,^58,59^ and a hope-based intervention was linked to increased pain tolerance.^60^

We acknowledge some limitations of our study. The study was entirely delivered remotely, which was our intention because the study was conceived during the COVID-19 lockdown period when access to allied professionals was limited. Given the remote delivery, all diagnoses and descriptions of pain areas are based on self-report, and though pain severity is a self-reported construct, we did not request access to medical records or referrals. Likewise, the frequency of app usage was based on self-report because we did not have access to mobile app tracking. Finally, the intervention was of relatively short duration considering the amount of time participants reported having been in pain. A longer trial may have resulted in more clinically meaningful changes in pain severity and interference scores. Similarly, the instructions for the intervention did not set a minimum time commitment per engagement with the app, nor did we require that participants engage with each modality. Given that certain activities require more cognitive effort or “work” than others, there may have been a bias toward choosing lower effort activities (e.g., education versus expressive writing). Future studies may consider setting time targets (in minutes) per day for app engagement, along with ensuring that a variety of exercises are attempted. Though we observed unequal drop out in the intervention compared to the usual care group, our mixed model analysis method estimated the outcomes, enabling an ITT analysis on all randomized participants.

We included two clinical tools to measure pain severity and interference, the BPI and PROMIS. They differ in terms of recall period, with a 24-h recall for the BPI and a 7-day recall for the PROMIS, and in terms of range (5-point vs. 11-point scale). We observed significant effects of the intervention for both severity and interference as measured by the BPI but did not observe significant effects on pain intensity on average as measured by the PROMIS. Scales (especially single-item scales) with more than 6 points have greater discriminatory power,^61^ and this may explain why small changes in pain intensity ratings are not reflected in the narrower 5-point scale.

Our sample reported moderate pain at baseline, with most participants reporting pain for >5 years (70%). Our sample was heterogeneous in terms of pain condition, household income, employment status, and age, which allows for generalizability across patient populations. It is important to note that our sample largely comprised participants identifying as women (82%), with a small representation of participants identifying as men (15%) or as non-binary (3%). Future studies might consider targeted recruitment of specific pain conditions, along with classifications into primary and secondary pain syndromes, to explore whether certain conditions derive greater benefits from a multimodal digital approach. Additionally, considering gender differences in the effectiveness of this type of intervention would be useful to inform implementation.

A remotely administered user-guided psychological intervention was effective at reducing self-reported physical (i.e., pain severity and interference with daily living), emotional (i.e., negative emotional states), and cognitive manifestations (i.e., beliefs about pain) of chronic pain. The mobile app can be accessible at any time, engagement can range from 1 min of calming breathing, to a 5-min micro-lesson about pain science, to 20- to 30-min meditations or brain training activities. Though there is an annual subscription cost, the cost is comparable to a single session of manual therapy, making this a potentially effective low-barrier treatment option that could be an immediate resource to patients.

## Supplementary Material

Supplemental Material

## Clinical Trials Registry

Trial was registered prior to starting participant recruitment (October 13, 2021), NCT No. 05090683, available at https://clinicaltrials.gov/ct2/show/NCT05090683.

## Data Deposition

The summary data are available at https://clinicaltrials.gov/ct2/show/NCT05090683. Additionally, the data sets generated and analyzed during the current study are available through the Open Science Framework at https://osf.io/b4dx6/files/osfstorage/65f8ed7118cee303731a296c. The DOI for the public data is: DOI https://10.17605/OSF.IO/B4DX6
