# Supplementary material for: Randomized controlled trial investigating the effectiveness of a multimodal mobile application for the treatment of chronic pain
Source: Can J Pain. 2024 Aug 19;8(1):2352399. doi: 10.1080/24740527.2024.2352399 (PMC11340744; doi:10.1080/24740527.2024.2352399)
Supplement: Supplemental Material [file UCJP_A_2352399_SM1400.docx]

**Randomized controlled trial investigating the effectiveness of a multimodal mobile application for the treatment of chronic or persistent pain**

**Supplementary Digital Content**

We report here the complete case analysis using our *a priori* analysis method: analysis of covariance (ANCOVA). The researcher carrying out the analyses described below was blinded to the groups (intervention vs. control).

**Methods**

***Statistical methods***

Normally distributed data that satisfied model assumptions were analyzed using ANCOVA to compare the effect of the intervention while taking baseline outcome data into account for each group (i.e., baseline data as the covariate, 6-week data as the dependent variable, and group (intervention *vs.* control) as the independent, between-subject factor). When data violated assumptions for ANCOVA, we applied Quade’s non-parametric ANCOVA.^1^ It is recommended that randomized controlled trials employ ANCOVA rather than paired t-tests or repeated measures ANOVA to control for baseline differences and increase power.^2,3^ We also provide data on differences from baseline using paired (dependent) t-tests for each group in the data table. Statistical significance was set at *p* < .05 for our co-primary outcomes which are derived from items on the same tool. Our secondary outcomes were significant at *p* < .05. Data for ANCOVA pairwise comparisons are reported in the results as *adjusted* mean ± standard error, unless otherwise noted. Effect sizes are reported as partial *eta-*square (η_p_^2^), where effect sizes are categorized as follows: .01 small; .06 medium, .14 large.^4^

We had pre-planned to compare descriptive data on medication use at baseline and at 6 weeks using dosage and frequency data, though post-hoc we simplified our analysis to include only dichotomous grouping based only on use (“yes” = reported use, or “no” = not reported) for the major sub-classes of medication reported. We compared frequencies between time (baseline *vs.* 6 weeks) and groups (intervention *vs.* usual care) using *Chi*-square (2x2) contingency tables.

**Results**

***Participant demographics***

Participant demographics are shown in Table S1. The average pain duration for the complete case sample was 13.3 years (sd = 11.1), ranging from 10 months to 43 years of persistent pain. Our remotely administered study included participants from nine countries with the majority residing in Canada (87.8%) and the United States (7%). Our inclusion criteria permitted a range of persistent pain conditions including migraine, musculoskeletal and visceral pain, and fibromyalgia. In the complete case sample, 59 (34%) participants reported receiving a formal diagnosis for a chronic pain condition, and of these 24 (41%) had co-occurring diagnoses. Frequencies of the four most reported diagnoses are shown in Table S1. Groups were stratified by gender and pain severity, and these variables did not differ between groups at baseline. Other variables, including age, pain duration, highest level of education, employment status, and annual household income did not differ significantly between groups (all *p* > .05).

***Primary Outcomes***

*BPI pain severity*

We checked assumptions for ANCOVA, and not all were satisfied. Standardized residuals were normally distributed and satisfied assumption of homoscedasticity based on visual inspection of scatterplots. There was one outlier (standardized residual > 3 standard deviations, 3.18), but given that the outlier appears to be an unusual value (pain score increased by 3 points) but does not appear to be due to error, we retained the data point. The data for the primary outcome, the BPI average pain severity score, violated assumptions of homogeneity of variance (HOV). We report ANCOVA results, and to verify robustness of the findings given the HOV violation, we also performed a non-parametric Quade’s ANCOVA.^1^  There was a significant difference in pain severity as measured by the BPI average score in the past 24 hours between groups at 6 weeks (ANCOVA: *F*(1, 168) = 12.44, *p* < .001, η_p_^2^ = .069). Non-parametric tests confirmed this result using Quade’s ANCOVA as the effect of the intervention was significant (*F*(1, 169) = 11.93, *p* = .001). After adjusting for baseline pain scores (adjusted means), BPI pain severity was lower following the intervention (4.52 ± 0.14) compared to the usual care group (5.16 ± 0.12) with a mean difference of -0.636 (95% CI, -0.992 to -0.280), *p* < .001) (see Table S2; Figure S1). At the 6-week follow up, 27.4% of individuals in the intervention group reported a minimal meaningful pain reduction of 2 points on the numerical rating scale^5^ compared to 10.2% of participants from the usual care group.

*BPI pain interference*

BPI pain interference data satisfied assumptions for ANCOVA, and there was a significant effect of the intervention compared to the usual care (*F*(1, 168) = 5.22, *p* = .024, η_p_^2^ = .030). After adjusting for baseline pain interference scores, BPI interference was lower following the intervention (4.84 ± 0.21) compared to the usual care group (5.47 ± 0.18) with a mean difference of -0.627 (95% CI, -1.170 to –0.085; Table S2; Figure S1).

***Secondary outcomes***

The complete case analysis of secondary outcomes revealed significant findings for pain catastrophizing, PROMIS interference, each of the DASS-21 subscales for depression, anxiety, and stress, and the SF-12 Quality of Life summary scores for physical health. There was little missing data overall (e.g., for PCS a total of 11 data points missing out of 4446 data points, or < .01%). But given that a single missing item in a sum score leads to a lower total score, we imputed the individual’s mode score when applicable. When an individual was missing 3 or more points from a single subscale in the DASS (7 items each) the score was not summed. Finally, for the SF-12 we imputed weighted mean scores (total missingness < .01%).

*Pain catastrophizing (PCS)*

Data from the PCS was skewed (at 6 weeks) and there was 1 outlier (at 3.29 SD from the mean) resulting from large changes in total score over time (+26 points). The ANCOVA result did not change with inclusion or exclusion of the outlier, but removal of the case restored normality (whereas no other transformations succeeded), so we removed the outlier. ANCOVA revealed a significant effect of treatment (*F*(1, 166) = 9.04, *p = .*003, η_p_^2^ = .052), with a lower adjusted mean following the intervention and a mean difference in pain catastrophizing of -3.76 (95% CI, -6.221 to -1.289).

*PROMIS*

PROMIS pain intensity on average scores were normally distributed (based on visual inspection of histograms, as Shapiro Wilk’s was not satisfied), with no outliers, and satisfied HOV and other ANCOVA assumptions. There was no significant difference in pain intensity as measured by the PROMIS 5-point scale with a 7-day recall period, *p* = .07. PROMIS interference scores violated assumptions for HOV and there was skewness in the usual care group. Non-parametric, Quade’s ANCOVA, revealed a significant treatment effect (*F*(1, 168) = 7.96, *p* = .005). Adjusted means (derived from ANCOVA) for PROMIS pain interference were lower following the intervention and a mean difference of -2.27 (95% CI, -4.02 to -0.52).

*DASS-21*

*DASS Anxiety.* Data from the DASS-21 subscales were slightly kurtotic and there was 1 outlier for DASS anxiety (+3.34 SD above mean). The case reported an 8-point increase in anxiety. Removal of the case restored normality and assumptions for ANCOVA were satisfied. Results were consistent with and without the inclusion of the outlier. ANCOVA for DASS-anxiety revealed a significant effect of the intervention (*F*(1, 164) = 11.27, *p* = .001, η_p_^2^ = .064). Adjusted means for anxiety were lower following the intervention compared to the usual care condition with a mean difference of -1.34 (95% CI, -2.135 to -0.554).

*DASS Depression.* Data for the depression subscale were normally distributed and there were no outliers. Data satisfied HOV but violated homogeneity of regression, so ANCOVA was not suitable (parametric or non-parametric). A repeated measures ANOVA was carried out as an alternative, and there was a significant time by group interaction (*F*(1, 167) = 7.313, *p* = .008, η_p_^2^ = .042). Participants reported lower scores in DASS-depression following the intervention compared to the usual care, mean difference of -1.462 (95% CI, -2.485 to -0.439).

*DASS Stress.* The final subscale of the DASS-21, DASS-stress data were skewed and had one outlier (+3.29 SD of mean) that reported increased stress by 10 points over the 6-week trial. Removal of the outlier did not restore normality and no transformations adequately satisfied assumptions for normality for DASS-stress. We therefore applied Quade’s ANCOVA, which revealed a significant intervention effect (*F*(1, 164) = 5.57, *p* = .019).

***SF-12 Quality of Life***

Both the physical and mental component summary scales (PC-12, MC-12) for the SF-12 satisfied all assumptions for ANCOVA. The intervention effect was significant for PC-12, at *F*(1, 165) = 3.98, *p* = .048, η_p_^2^ = .024). The intervention group reported slightly higher physical health scores, with an adjusted mean difference of +2.32 (95% CI: 0.024 to 4.616). There was no significant effect of the intervention for the mental health scores (*p* = .86). Both groups report physical component scores well below population mean (below 25^th^ percentile general US population), and mental component scores fall around the mean in both groups.^6^

***Medication use***

The data for completers revealed no differences in medication usage between groups at baseline, save reported use of anti-convulsant in usual care *vs*. intervention group (𝜒^2^= 4.18, *p* = .041). The only within group change over time was in the usual care group with a reported increase in the use of non-opioid analgesics from baseline to 6 weeks (𝜒^2^ = 3.89, *p* = .049). Finally, between groups at 6 weeks there was a difference in CNS depressant use, with the usual care group reporting greater use at 6 weeks compared to the intervention group (Fisher’s exact: *p* = .029), with no difference between groups at baseline (*p* = 1.0). Data are shown in Table S3.

**References**

1. Quade D. Rank Analysis of Covariance. *J Am Stat Assoc*. 1967;62(320):1187-1200. doi:10.1080/01621459.1967.10500925

2. Borm GF, Fransen J, Lemmens WAJG. A simple sample size formula for analysis of covariance in randomized clinical trials. *J Clin Epidemiol*. 2007;60(12):1234-1238. doi:10.1016/j.jclinepi.2007.02.006

3. Egbewale BE, Lewis M, Sim J. Bias, precision and statistical power of analysis of covariance in the analysis of randomized trials with baseline imbalance: A simulation study. *BMC Med Res Methodol*. 2014;14(1). doi:10.1186/1471-2288-14-49

4. Cohen J. *Statistical Power Analysis for the Behavioral Sciences* . 2nd ed. Lawrence Erlbaum Associates.; 1988.

5. Dworkin RH, Turk DC, Wyrwich KW, et al. Interpreting the Clinical Importance of Treatment Outcomes in Chronic Pain Clinical Trials: IMMPACT Recommendations. *J Pain*. 2008;9(2):105-121. doi:10.1016/j.jpain.2007.09.005

6. Ware J, Kosinski M, Keller S. *SF-12: How to Score the SF-12 Physical and Mental Health Summary Scales*.; 1995.

**Table S1**

***Participant demographics at baseline for study completers***

| Characteristic | Wait-listed usual care (n = 98) | Intervention (*n* = 73) |
| --- | --- | --- |
| *Demographic features* |  |  |
| Age (years), mean(SD) | 47.0 (13.0) | 46.8 (13.4) |
| Gender, *n(%)* |  |  |
| Woman | 82 (83.7) | 59 (80.8) |
| Man | 14 (14.3) | 11 (15.1) |
| Non-binary | 2 (2.0) | 2 (2.7) |
| Education, *n*(%) |  |  |
| High school or less | 10 (10.2) | 8 (10.9) |
| Trade or diploma | 32 (32.6) | 20 (27.4) |
| Bachelor degree or higher | 54 (55.1) | 44 (60.3) |
| Household income, *n*(%) |  |  |
| < $34 999 | 26 (26.5) | 17 (23.3) |
| $35 000 to $49 999 | 3 (3.1) | 10 (13.7) |
| $50 000 to $74 999 | 20 (20.4) | 14 (19.2) |
| $74 999 to $99 999 | 19 (19.4) | 10 (13.7) |
| > $100 0000 | 26 (26.5) | 20 (27.4) |
| Employment status, *n*(%) |  |  |
| Unable to work/on Disability | 26 (26.5) | 17 (23.3) |
| Employed (full and part time) | 47 (48.0) | 37 (50.7) |
| Other (student, homemaker) | 10 (10.2) | 8 (11.0) |
| Retired | 13 (13.3) | 10 (13.7) |
| *Clinical features*, mean(SD) |  |  |
| Pain duration (years) | 14.9 (14.5) | 12.4 (10.7) |
| BPI on average | 5.36 (1.5) | 5.33 (1.5) |
| Diagnosis^a^, *n*(%) |  |  |
| No diagnosis | 64 (65.8) | 48 (65.3) |
| Fibromyalgia | 20 (20.4) | 15 (20.5) |
| Myalgic Encephalomyelitis | 6 (6.1)^b^ | 4 (4.1)^b^ |
| Osteoarthritis | 11 (11.2) | 7 (9.6) |
| Migraine | 7 (7.1) | 5 (6.8) |
| Other (not including above) | 6 (6.1) | 6 (8.2) |

*Notes.* Values are *n*(%) for categorical data, mean(SD) for continuous data. Baseline comparisons between groups employed independent *t-*test for continuous variables and *chi* square for frequencies. There were no significant differences between groups for any of the variables.

^a^Counts for diagnoses are not mutually exclusive as many (41% of those with formal diagnoses) reported multiple diagnoses.

^b^all participants reporting myalgic encephalomyelitis also reported fibromyalgia.

**Table S2**

***Outcome variables at baseline and 6 weeks for complete cases***

|  | Usual care group, *n* = 98 | |  | | Intervention group, *n* = 73 | |  |  |  |  | Main analyses: Between subjects | |
| --- | --- | --- | --- | --- | --- | --- | --- | --- | --- | --- | --- | --- |
|  |  | | Within subjects^a^ | |  | |  | Within subjects at 6 weeks^a^ | | Within subjects at 12 weeks^b^ | Baseline to 6-weeks |  |
| Outcome | Baseline | 6-weeks | *n* | *p* | Baseline | 6-weeks | 12 weeks  (*n* = 65) | *n* | *p* | p | *p*^c^ |  |
| BPI severity | 5.35 (1.51) | 5.16 (1.44) | 98 | .063 | 5.33 (1.47) | 4.51 (1.74) | 4.60 (1.70) | 73 | <.001 | <.001 | .001^c^ |  |
| BPI interference | 6.05 (2.26) | 5.49 (2.30) | 98 | .001 | 5.97 (2.14) | 4.81 (2.32) | 4.85 (2.49) | 73 | <.001 | <.001 | .024 |  |
| PROMIS intensity | 3.22 (0.60) | 3.08 (0.61) | 97 | .032 | 2.99 (0.59) | 2.81 (0.59) | 2.74 (0.54) | 73 | .015 | <.001 | .07 |  |
| PROMIS interference | 29.01 (7.46) | 19.63 (8.28) | 97 | <.001 | 28.22 (7.30) | 16.71 (8.36) | 24.34 (7.54) | 73 | <.001 | <.001 | .005^c^ |  |
| Pain catastrophizing | 23.13 (11.30) | 20.38 (12.00) | 97 | < .001 | 22.55 (12.22) | 15.92 (11.48) | 16.46 (12.83) | 73 | <.001 | <.001 | .003 |  |
| DASS-depression | 14.52 (9.84) | 14.85 (10.75) | 96 | .621 | 14.57 (9.52) | 11.97 (9.09) | 12.65 (9.98) | 73 | .004 | .042 | .008 |  |
| DASS-anxiety | 8.64 (6.92) | 8.94 (6.93) | 94 | .336 | 9.89 (7.85) | 7.31 (7.73) | 8.31 (7.43)^d^ | 72 | <.001 | .013 | .001 |  |
| DASS-stress | 15.35 (8.33) | 15.46 (8.63) | 96 | .882 | 16.54 (8.78) | 13.63 (8.14) | 15.17 (9.35)^d^ | 71 | .003 | .026 | .019^a^ |  |
| SF-12 PHC | 21.60 (9.58) | 23.89 (11.36) | 95 | .002 | 25.21 (11.07) | 29.27 (11.36) | 27.66 (10.34) | 73 | <.001 | .005 | .048 |  |
| SF-12 MHC | 49.18 (6.74) | 50.05 (6.97) | 95 | .111 | 47.83 (5.47) | 49.22 (6.39) | 50.20 (7.62) | 73 | .032 | .005 | .86 |  |

*Notes.* Values are mean (SD). Data shown above have not been transformed. Sample sizes vary due to missing data for certain variables.

^a^ *p* value and sample sizes for the within-group differences (baseline to 6 weeks).

^b^p value for the within-group differences in the intervention group only (baseline to 12 weeks).

^c^*p* value for the between-group differences over time (analysis of covariance with baseline data value as covariate).

^c^Quade’s ANCOVA. ^d^data for *n* = 64.

**Table S3**. Pain medication usage at baseline and post-intervention for study completers.

|  | Usual care (*n* = 98) | | Intervention (*n* = 73) | |
| --- | --- | --- | --- | --- |
| Description of medication use, *n*(%)^a^ | Baseline | 6-weeks | Baseline | 6-weeks |
| Are you taking any medication for pain? |  |  |  |  |
| Yes | 89 (90.8) | 87 (88.8) | 64 (87.7) | 61 (83.6) |
| No | 9 (9.2) | 11 (11.2) | 9 (12.3) | 12 (16.4) |
| Are you taking daily medication for pain? |  |  |  |  |
| Yes | 67 (68.4) | 67 (68.4) | 48 65.8 | 42 (57.5) |
| No | 31 (31.6) | 31 (31.6) | 25 34.2 | 31 (42.5) |
| Non-opioid analgesia |  |  |  |  |
| Reported | 59 (60.2)^b^ | 72 (73.5)^b^ | 50 (68.4) | 51 (69.9) |
| Not reported | 39 (39.8) | 26 (26.5) | 23 (31.5) | 22 (30.1) |
| Opioid analgesia |  |  |  |  |
| Reported | 29 (29.6) | 30 (30.6) | 13 (17.8) | 14 (19.2) |
| Not reported | 69 (70.4) | 68 (69.4) | 60 (82.2) | 59 (80.8) |
| Anti-depressant |  |  |  |  |
| Reported | 27 (27.6) | 32 (32.7) | 21 (28.8) | 18 (24.7) |
| Not reported | 71 (72.4) | 66 (67.3) | 52 (71.2) | 55 (75.3) |
| Anti-convulsant |  |  |  |  |
| Reported | 31 (31.6)^c^ | 26 (26.5) | 13 (17.8)^c^ | 17 (23.3) |
| Not reported | 67 (68.4) | 72 (73.5) | 60 (82.2) | 56 (76.7) |
| CNS depressant |  |  |  |  |
| Reported | 9 (9.2) | 9 (9.2)^d^ | 6 (8.2) | 1 (1.4)^d^ |
| Not reported | 89 (90.8) | 89 (90.8) | 67 (91.8) | 72 (98.6) |
| Other meds related to pain |  |  |  |  |
| Reported | 9 (9.2) | 11 (11.2) | 9 (12.3) | 12 (16.4) |
| Not reported | 89 (90.8) | 87 (88.8) | 67 (87.7) | 61 (83.6) |

*Notes.* There were no differences in frequency of medication use or drug classifications between groups at baseline (between group comparisons). There were no differences in medication use within groups over time (within group comparisons). Pearson’s *Chi-*square test employed for cell frequencies > 5, Fisher Exact test if cells < 5. All *p* > .05 unless otherwise noted.

^a^Counts for medications are not mutually exclusive as many reported multiple medications.

^b, c, d^represent differences (*p* < .05) between pairs of cell frequencies.

*

**

Figure S1. Change scores for Brief Pain Inventory Primary Outcomes from baseline to 6 weeks *(n* = 73 for intervention; *n* = 98 for usual care)*.* BPI severity reflects pain on average over the past 24 hours. BPI interference is an average score. **p* < .05;***p* < .001 for difference between groups. Error bars represent standard error of change score.
